# Effects of resistance training on metabolic and cardiovascular responses to a maximal cardiopulmonary exercise test in Parkinson`s disease

**DOI:** 10.31744/einstein_journal/2021AO5940

**Published:** 2021-04-09

**Authors:** Hélcio Kanegusuku, Tiago Peçanha, Carla Silva-Batista, Roberto Sanches Miyasato, Natan Daniel da Silva, Marco Túlio de Mello, Maria Elisa Pimentel Piemonte, Carlos Ugrinowitsch, Cláudia Lúcia de Moraes Forjaz

**Affiliations:** 1 Hospital Israelita Albert Einstein São PauloSP Brazil Hospital Israelita Albert Einstein, São Paulo, SP, Brazil.; 2 Universidade de São Paulo São PauloSP Brazil Universidade de São Paulo, São Paulo, SP, Brazil.; 3 Universidade Federal de Minas Gerais Belo HorizonteMG Brazil Universidade Federal de Minas Gerais, Belo Horizonte, MG, Brazil.

**Keywords:** Parkinsonian disorders, Cardiovascular abnormalities, Oxygen consumption, Exercise test, Resistance training

## Abstract

**Objective:**

To evaluate the effects of resistance training on metabolic and cardiovascular responses during maximal cardiopulmonary exercise testing in patients with Parkinson’s disease.

**Methods:**

Twenty-four patients with Parkinson’s disease (modified Hoehn and Yahr stages 2 to 3) were randomly assigned to one of two groups: Control or Resistance Training. Patients in the Resistance Training Group completed an exercise program consisting of five resistance exercises (two to four sets of six to 12 repetitions maximum per set) twice a week. Patients in the Control Group maintained their usual lifestyle. Oxygen uptake, systolic blood pressure and heart rate were assessed at rest and during cycle ergometer-based maximal cardiopulmonary exercise testing at baseline and at 12 weeks. Assessments during exercise were conducted at absolute submaximal intensity (slope of the linear regression line between physiological variables and absolute workloads), at relative submaximal intensity (anaerobic threshold and respiratory compensation point) and at maximal intensity (maximal exercise). Muscle strength was also evaluated.

**Results:**

Both groups had similar increase in peak oxygen uptake after 12 weeks of training. Heart rate and systolic blood pressure measured at absolute and relative submaximal intensities and at maximal exercise intensity did not change in any of the groups. Muscle strength increased in the Resistance Training but not in the Control Group after 12 weeks.

**Conclusion:**

Resistance training increases muscle strength but does not change metabolic and cardiovascular responses during maximal cardiopulmonary exercise testing in patients with Parkinson’s disease without cardiovascular comorbidities.

## INTRODUCTION

Parkinson’s disease (PD) is a progressive neurodegenerative disorder characterized by nigrostriatal dopaminergic system dysfunction and resultant motor symptoms, such as bradykinesia, resting tremor, rigidity and postural instability.^(^^1^^,^^2^^)^ Besides motor symptoms, PD patients also tend to have metabolic and cardiovascular dysfunctions either at rest^(^^3^^–^^6^^)^ or during stressful events such as exercise.^(^^7^^–^^10^^)^ Attenuated metabolic and cardiovascular responses in submaximal and maximal cardiopulmonary exercise testing have recently been reported in patients with PD.^(^^7^^)^ Blunted responses to exercise may increase cardiovascular risk in these patients.^(^^11^^,^^12^^)^ Hence the significance of investigating strategies to offset such impairments.

Resistance training (RT) is recommended to PD patients in order to improve muscle strength, functionality (*e.g*., walking capacity) and quality of life.^(^^13^^)^ Resistance training has also been shown to improve cardiac autonomic dysfunction at rest and during stressful conditions such as orthostatic stress in this population.^(^^14^^)^ These findings suggest RT may also enhance cardiovascular responses to exercise. However, this hypothesis remains to be confirmed.

Resistance training has been shown to increase peak oxygen uptake (VO_2peak_) in patients suffering from other conditions (cognitive impairment, hemiparesis, etc).^(^^15^^,^^16^^)^ Still, in PD patients in particular, RT failed to induce changes in VO_2peak_^(^^17^^,^^18^^)^ and RT-related effect remains to be confirmed. However, assessment of responses to submaximal exercise (*i.e*., responses at absolute submaximal workloads and ventilatory thresholds) is even more relevant in these patients, since these responses mirror effort intensity associated with daily activities.^(^^19^^)^ Fernández-Lezaun et al., reported improvement of responses to submaximal exercise in older adults without PD following RT.^(^^20^^)^ Enhanced cardiovascular responses to submaximal RT may improve the quality of life of these patients.^(^^21^^)^ Despite the relevance of this topic, to the best of our knowledge the effects of training on responses to submaximal exercise have not been investigated in PD patients to date.

This study was designed to test the hypothesis that RT improves responses obtained at submaximal intensities during maximal cardiopulmonary exercise testing in patients with PD without changing maximal metabolic (*i.e*., VO_2_) and cardiovascular (*i.e*., heart rate – HR and systolic blood pressure – SBP) parameters.

## OBJECTIVE

To evaluate the effects of resistance training on metabolic and cardiovascular responses during maximal cardiopulmonary exercise testing in patients with Parkinson’s disease.

## METHODS

### Subjects

Volunteers were recruited from *Associação Brasil Parkinson*. Inclusion criteria were as follows: ≥50 years of age; stages 2 to 3 of the modified Hoehn and Yahr scale;^(^^22^^)^ no other neurological disorder, hypertension or cardiovascular disease diagnosis; not taking medications with potential direct cardiovascular effects except for those required to treat PD; no limitations to engage in RT. Patients who experienced changes of type or dose of regular medications during the study, engaged in additional physical exercise programs or attended to less than 80% of RT sessions were excluded.

This paper was based on data extracted from a larger study approved by the Ethics Committee of the *Faculdade de Educação Física e Esporte* of *Universidade de São Paulo* (2011/42) and registered at Brazilian Clinical Trials Registry (RBR-5YC53K). All patients read and signed an informed consent form prior to enrollment. Findings derived from the analysis RT effects on resting conditions have been published.^(^^14^^)^This paper specifically addresses responses during exercise.

### Experimental design

This prospective, randomized controlled study with parallel design was conducted at the *Faculdade de Educação Física e Esporte* of *Universidade de São Paulo*, between February 2012 and March 2015. Patients with PD were randomly allocated to one of two groups: Control Group (CG) or Resistance Training Group (RTG), as previously described.^(^^14^^)^

Physiological responses during maximal cardiopulmonary exercise testing were assessed at baseline (beginning of the study) and at 12 weeks. In the RTG, post-intervention assessments were carried out at least 48 hours after the last training session. Patients were assessed in the “on” state *(i.e*., action phase of medication).

### Experimental procedures

Detailed description of maximal cardiopulmonary exercise testing procedures have been given elsewhere.^(^^7^^)^Tests were conducted on a cycle ergometer (Lode, Corival, Netherlands) by a physician with extensive experience (more than 10 years) in maximal exercise testing. Fifteen to twenty minutes prior to testing, patients were familiarized with the cycle ergometer by pedaling at a comfortable intensity for 2 to 3 minutes. Patients were then allowed to rest until cardiovascular parameters returned to baseline and tested. Individualized ramp protocols were selected to induce fatigue within 8 to 12 minutes of test start. These involved increments ranging from 3 to 15 watts per minute according PD severity and level of physical conditioning. Pedaling frequency ranged from 50 to 60rpm. Tests were discontinued whenever subjects were unable to maintain pedaling frequency. The same ramp increments were used in baseline and 12-week assessments.

Heart rate was recorded at 30-second intervals via continuous 12-lead echocardiogram monitoring (CardioPerfect^®^, ST 2001, Netherlands). Auscultatory blood pressure was measured by a blinded technician every 2 minutes using a mercury sphygmomanometer. Respiratory gas exchange was measured by breath-by-breath analysis using a metabolic cart (Medical Graphics Corporation, CPX/D, United States) and data collected at 30-second intervals averaged.

Peak HR, SBP and VO_2_ were defined as the highest value obtained during the exercise phase of the test and corresponded to maximal test responses. Responses to submaximal relative intensities were assessed at ventilatory thresholds (*i.e*., anaerobic threshold – AT – and respiratory compensation point – RCP)^(^^23^^,^^24^^)^ determined by two independent experts, with discrepancies solved by consensus. Responses to submaximal absolute intensities were assessed by comparing the slope of the linear regression line between physiological parameters (VO_2_, HR and SBP values) recorded at baseline and after 12 weeks of exercise practice.^(^^25^^)^ Regression was based on values measured during exercise. Individual linear regression slope lines were used in the analysis.

### Interventions

The RT program consisted of two exercise sessions per week with a rest period of 3 to 4 days in-between. Training sessions were supervised by an experienced strength coach. The RT program included lower limb (horizontal leg press, squat and rotary calf) and upper limb (lat pulldown and chest press) resistance exercises. Training load increased progressively from two to four sets of six to 12 repetition maximum (RM), as detailed elsewhere.^(^^14^^)^ Training load increments were as follows: first and second weeks, two sets of 10-12 RM; third and fourth weeks, three sets of 10-12 RM); fifth and sixth week, three sets of 8-10 RM; seventh to tenth week, four sets of 8-10 RM; 11^st^ and 12^nd^ weeks, four sets of 6-8 RM. The rest period between exercises and sets was 2 minutes. Workload increased progressively throughout the intervention period. Load increments were introduced whenever patients were able to execute two consecutive sessions with the same load.

Patients in the CG were instructed to maintain their usual lifestyle during the experimental period and were evaluated at baseline and at 12 weeks only.

### Muscle strength

Resistance training efficacy was estimated according to muscle strength. After two familiarization sessions 48 hours apart, muscle strength was assessed using the 1RM test in the leg press exercise, *as per* the Brown et al., protocol^(^^26^^)^ and details given elsewhere.^(^^14^^)^ Muscle strength was reassessed at 12 weeks.

### Statistical analysis

Data normality and homogeneity were investigated using the Shapiro-Wilk and Levene test respectively. Data were submitted to logarithmic transformation as needed (*i.e*., HR_peak_, HR_slope_ and VO_2slope_). Patient characteristics were compared between groups using the *t* or the χ^2^ test. Two-way mixed design analysis of variance (ANOVA - *análise de variância*) using group (CG *versus* RTG) as a between main factor and time (baseline and 12 weeks) as a within main factor was conducted to examine the effects of RT. Whenever F-values were significant, the Newman-Keuls *post-hoc* test was used for multiple comparisons. Variables (*i.e*., SBP) that differed between groups at baseline were submitted to covariance analysis (ANCOVA - *análise de covariância*) using baseline values as covariates. Effect size (ES) was calculated for each outcome using Cohen’s d.^(^^27^^)^ Effect size was categorized as small (ES≤0.49), medium (ES 0.50-0.79) or large (ES≥0.80). The level of significance was set at p<0.05. Data were expressed as means±standard deviation.

## RESULTS

**Figure 1:**
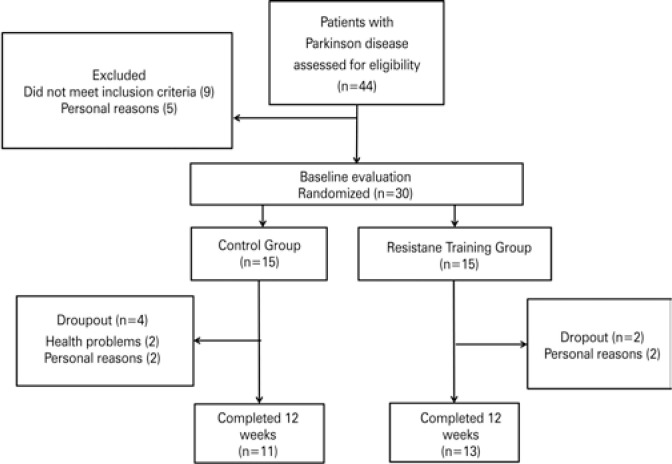
Flowchart of participants

Forty-four PD patients signed the informed consent form. Of these, 30 were randomly allocated to the RTG (n=15) or the CG (n=15). Four patients in the CG and two patients in the RTG dropped out of the study during the intervention period. A total of 24 patients completed the study (11 and 13 patients, CG and RTG, respectively) and had their data analysed (Figure 1).

Patient characteristics were similar between groups at baseline (Table 1). Patients in the RTG completed more than 90% of training sessions. Lower limb strength remained unchanged in the CG (90±26kg *versus* 82±26kg) but increased significantly in RTG (90±24kg *versus* 108±29kg) after 12 weeks of RT. Muscle strength differed significantly between groups at 12 weeks (F _[1,22]_ = 83.159; p<0.01).

**Table 1:** Characteristics of patients with Parkinson Disease allocated to the Control or the Resistance Training Group

		Control Group (n=11)	Resistance Training Group (n=13)	p value
Physical characteristics					
	Sex, male/ female	8/ 3	11/ 2	0.48
	Age, years	62±9	67±8	0.12
	Body mass index, kg/m²	27.1±3.4	25.9±3.6	0.43
PD characteristics					
	Disease duration, years	9±4	9±4	0.94
	Hoehn & Yahr modified, stage 2/2.5/3	5/ 2/ 4	6/ 2/ 5	0.98
Medication use					
	Levodopa/carbidopa	11	11	0.18
	Dopamine agonist	6	5	0.43
	Amantadine	4	6	0.63
	Selegiline	2	3	0.77

Data expressed as n or mean±standard deviation.

PD: Parkinson’s disease.

Baseline VO_2peak_ did not differ between groups. However, both groups experienced similar significant increase in VO_2peak_ after the intervention (F_[1,22]_ = 0.0338; p=0.86 and p<0.01, interaction effect and main effect of time respectively) (Table 2). Resting VO_2peak_ AT and RCP were also similar between groups at baseline and remained unchanged over time. Likewise, peak VO_2_ increase according to workload increments (slope line between VO_2_ and workload) was similar between groups and remained unchanged over time.

**Table 2:** Metabolic and cardiovascular responses assessed at rest and at submaximal and maximal intensities during cardiopulmonary exercise testing conducted at baseline and at 12 weeks with Parkinson disease patients in the Control and the Resistance Training group

	Control Group		ES	Resistance Training Group		ES	F-values	p value
	Baseline	12 weeks		Baseline	12 weeks			
Peak workload (watts	107±54	107±58	0.01	93±37	96±38	0.09	F_[1,22]_=0.5877	0.45
VO_2slope_, mL.kg^−1^.min^−1^/watt†	−1.03±0.38	−0.86±0.14	0.75	−0.90±0.21	−0.88±0.12	0.05	F_[1,22]_=1.1013	0.31
VO_2AT_, mL.kg.^−1^min^−1^	9.7±2.1	9.7±2.2	0.00	10.3±2.3	10.3±2.8	0.02	F_[1,22]_=0.0090	0.93
VO_2RCP_, mL.kg.^−1^min^−1^	14.4±3.5	13.8±3.6	0.03	14.6±2.4	15.7±3.0	0.40	F_[1,20]_=1.7885	0.20
VO_2peak_, mL.kg.^−1^min^−1^	17.8±5.5	18.6±5.8*	0.14	18.0±4.2	18.8±4.4*	0.20	F_[1,22]_=0.0338	0.86
HR_rest_, bpm	76±8	76±11	0.06	73±8	72±10	0.16	F_[1,22]_=0.2541	0.62
HR_slope_, bpm/watt†	−0.28±0.19	−0.24±0.29	0.18	−0.38±0.17	−0.47±0.26	0.44	F_[1,22]_=3.3466	0.08
HR_AT_, bpm	110±13	108±12	0.17	98±17	99±17	0.07	F_[1,22]_=0.6767	0.42
HR_RCP_, bpm	126±14	124±15	0.13	114±22	118±22	0.19	F_[1,20]_=2.0427	0.17
HR_peak_, bpm†	2.16±0.06	2.15±0.06	0.24	2.10±0.09	2.10±0.10	0.03	F_[1,22]_=2.3841	0.14
SBP_rest_, mmHg	113±13	107±12	0.47	122±18#	119±14#	0.16	F_[1,22]_=0.1535	0.70
SBP_slope_, mmHg/watt	0.72±0.64	0.95±0.80	0.33	0.45±0.25	0.57±0.25	0.50	F_[1,18]_=0.4273	0.52
SBP_AT_, mmHg	139±17	132±15	0.41	137±16	144±19	0.43	F_[1,22]_=3.1499	0.09
SBP_RCP_, mmHg	156±22	148±18	0.39	149±15	158±18	0.58	F_[1,20]_=5.6330	0.03
SBP_peak_, mmHg	171±23	170±25	0.10	160±20	167±20	0.37	F_[1,22]_=1.6963	0.21

Results expressed as mean±standard deviation.

* significantly different from baseline (p<0.05);

† logarithm;

# significantly different from the C Group (p<0.05).

EF: effect size; VO_2_: oxygen uptake; AT: anaerobic threshold; RCP: respiratory compensation point; HR: heart rate; SBP: systolic blood pressure.

Heart rate measured at rest, AT, RCP and maximal exercise, and HR slope increment per watt during testing were similar between groups and remained unchanged after 12 weeks of RT. Likewise, SBP measured at, RCP and maximal exercise, and SBP slope increment per watt during testing did not differ between groups and did not change after 12 weeks of RT.

## DISCUSSION

The main finding of this study was that RT increased lower limb muscle strength but had no impact on metabolic and cardiovascular responses obtained at maximal or absolute and relative submaximal intensities during maximal cardiopulmonary exercise testing in patients with PD.

Lower limb muscle strength increase in RTG relative to the CG emphasizes the effectiveness of the training protocol employed in this study. Similar findings have been reported in previous studies,^(^^17^^,^^18^^)^ in which lower limb strength also increased after RT. Neuroplastic changes in the primary motor cortex and neural adaptation to RT may explain the initial increase in lower limb strength in these patients.^(^^28^^–^^30^^)^ Such strength gains may be of clinical significance in this population, given muscle mass and strength losses are correlated with poorer quality of life in patients with PD.^(^^31^^)^

Significant similar VO_2peak_ increase at 12 weeks in both groups indicates improvements cannot be attributed to the RT program. In fact, the increase observed in the CG suggests a learning effect of multiple tests. A previous study^(^^32^^)^ with PD patients submitted to three maximal cardiopulmonary exercise tests on different days revealed significant increases in VO_2peak_ between the first and the second test. Patients in this study may have experienced similar effects.

The lack of effect of RT on VO_2peak_ is in keeping with previous studies that also failed to detect changes in VO_2peak_ in response to RT in patients with PD.^(^^17^^,^^18^^)^ This finding is also consistent with a previous study conducted by this research group,^(^^25^^)^ in which VO_2peak_ remained unchanged after 16 weeks of resistance and power training in healthy elderly. In contrast, studies conducted with other populations of adult patients with neurological impairments or chronic neurologic diseases (cognitive impairments or chronic hemiparesis) reported VO_2_ increase after RT.^(^^15^^,^^16^^)^ This discrepancy may reflect different pathophysiological changes in different neurological diseases and their respective impact on patient response to RT. Therefore, it could be argued that, at least in patients with mild to moderate stage PD, RT has no effect on VO_2peak_.

A differential of this study was the investigation of submaximal exercise parameters which are less impacted by motor disabilities associated with PD.^(^^33^^)^ For this purpose, patients were assessed at both absolute and relative submaximal exercise intensities. Responses to absolute submaximal intensity were assessed according to metabolic and cardiovascular changes in response to workload increments, whereas responses to relative submaximal intensity were assessed at AT and RCP. Contrary to the research hypothesis, RT had no impact on VO_2_, HR or SBP responses at absolute and relative submaximal intensities. This finding is congruent with a previous study addressing responses to RT at absolute and relative submaximal intensities in subjects without PD.^(^^25^^)^ Therefore, is spite selection of parameters which are less affected by the disease-related limitations, this study failed to demonstrate significant impacts of RT on responses during progressive exercise testing.

Enhanced metabolic and cardiovascular responses during maximal cardiopulmonary exercise testing are associated with better quality of life in patients with PD. Such responses have been obtained with aerobic training in this population.^(^^18^^,^^34^^)^ Still, RT can improve muscle strength, functionality (*e.g*., walking capacity) and quality of life in patients with PD.^(^^17^^,^^18^^,^^26^^,^^28^^,^^29^^,^^33^^,^^35^^)^ Findings of this study support recommendations of combined aerobic and strength training in exercise programs designed for patients in the mild to moderate stages of PD.

### Study limitations

This study has some limitations. Exclusion of patients diagnosed with hypertension or any other cardiovascular disease limited sample size. However, such exclusions were deemed important to tease out the effects of RT on metabolic and cardiovascular changes inherent to PD. Also, selected patients were at stages 2 to 3 of the modified Hoehn & Yahr scale. Therefore, findings may not apply to patients in other stages of PD.

## CONCLUSION

Twelve weeks of resistance training improves lower limb muscle strength but does not affect metabolic or cardiovascular responses obtained at submaximal and maximal intensities during maximal cardiopulmonary exercise testing in patients with Parkinson’s disease with no cardiovascular comorbidities.

## References

[1] 1. Jankovic J. Parkinson’s disease: clinical features and diagnosis. J Neurol Neurosurg Psychiatry. 2008;79(4):368-76. Review. 10.1136/jnnp.2007.131045 18344392

[2] 2. Poewe W, Mahlknecht P. The clinical progression of Parkinson’s disease. Parkinsonism Relat Disord. 2009;15 Suppl 4:S28-32. Review. 10.1016/S1353-8020(09)70831-4 20123553

[3] 3. Barbic F, Perego F, Canesi M, Gianni M, Biagiotti S, Costantino G, et al. Early abnormalities of vascular and cardiac autonomic control in Parkinson’s disease without orthostatic hypotension. Hypertension. 2007;49(1):120-6. 10.1161/01.HYP.0000250939.71343.7c 17101845

[4] 4. Goldstein DS. Dysautonomia in Parkinson disease. Compr Physiol. 2014;4(2): 805-26. Review. 10.1002/cphy.c130026 PMC4222515 24715569

[5] 5. Kaufmann H, Goldstein DS. Autonomic dysfunction in Parkinson disease. Handb Clin Neurol. 2013;117:259-78. Review. 10.1016/B978-0-444-53491-0.00021-3 24095131

[6] 6. Magerkurth C, Schnitzer R, Braune S. Symptoms of autonomic failure in Parkinson’s disease: prevalence and impact on daily life. Clin Auton Res. 2005;15(2):76-82. 10.1007/s10286-005-0253-z 15834763

[7] 7. Kanegusuku H, Silva-Batista C, Peçanha T, Nieuwboer A, Silva ND Jr, Costa LA, et al. Blunted maximal and submaximal responses to cardiopulmonary exercise tests in patients with Parkinson disease. Arch Phys Med Rehabil. 2016;97(5):720-5. 10.1016/j.apmr.2015.12.020 26780469

[8] 8. Low DA, Vichayanrat E, Iodice V, Mathias CJ. Exercise hemodynamics in Parkinson’s disease and autonomic dysfunction. Parkinsonism Relat Disord. 2014;20(5):549-53. 10.1016/j.parkreldis.2014.02.006 24637120

[9] 9. Sabino-Carvalho JL, Teixeira AL, Samora M, Daher M, Vianna LC. Blunted cardiovascular responses to exercise in Parkinson’s disease patients: role of the muscle metaboreflex. J Neurophysiol. 2018;120(4):1516-24. 10.1152/jn.00308.2018 29947592

[10] 10. Miyasato RS, Silva-Batista C, Peçanha T, Low DA, de Mello MT, Piemonte ME, et al. Cardiovascular responses during resistance exercise in patients with Parkinson disease. PM R. 2018;10(11):1145-52. 10.1016/j.pmrj.2018.04.009 29753113

[11] 11. Lauer MS, Francis GS, Okin PM, Pashkow FJ, Snader CE, Marwick TH. Impaired chronotropic response to exercise stress testing as a predictor of mortality. JAMA. 1999;281(6):524-9. 10.1001/jama.281.6.524 10022108

[12] 12. Oliveira RB, Myers J, Araújo CG, Abella J, Mandic S, Froelicher V. Maximal exercise oxygen pulse as a predictor of mortality among male veterans referred for exercise testing. Eur J Cardiovasc Prev Rehabil. 2009;16(3):358-64. 10.1097/HJR.0b013e3283292fe8 19357518

[13] 13. Falvo MJ, Schilling BK, Earhart GM. Parkinson’s disease and resistive exercise: rationale, review, and recommendations. Mov Disord. 2008;23(1):1-11. Review. 10.1002/mds.21690 17894327

[14] 14. Kanegusuku H, Silva-Batista C, Peçanha T, Nieuwboer A, Silva ND Jr, Costa LA, et al. Effects of progressive resistance training on cardiovascular autonomic regulation in patients with Parkinson disease: a randomized controlled trial. Arch Phys Med Rehabil. 2017;98(11):2134-41. 10.1016/j.apmr.2017.06.009 28705551

[15] 15. Ivey FM, Prior SJ, Hafer-Macko CE, Katzel LI, Macko RF, Ryan AS. Strength training for skeletal muscle endurance after stroke. J Stroke Cerebrovasc Dis. 2017;26(4):787-94. 10.1016/j.jstrokecerebrovasdis.2016.10.018 PMC5947878 27865696

[16] 16. Mavros Y, Gates N, Wilson GC, Jain N, Meiklejohn J, Brodaty H, et al. Mediation of cognitive function improvements by strength gains after resistance training in older adults with mild cognitive impairment: outcomes of the Study of Mental and Resistance Training. J Am Geriatr Soc. 2017;65(3):550-9. 10.1111/jgs.14542 28304092

[17] 17. Demonceau M, Maquet D, Jidovtseff B, Donneau AF, Bury T, Croisier JL, et al. Effects of twelve weeks of aerobic or strength training in addition to standard care in Parkinson’s disease: a controlled study. Eur J Phys Rehabil Med. 2017;53(2):184-200. 10.23736/S1973-9087.16.04272-6 27585055

[18] 18. Shulman LM, Katzel LI, Ivey FM, Sorkin JD, Favors K, Anderson KE, et al. Randomized clinical trial of 3 types of physical exercise for patients with Parkinson disease. JAMA Neurol. 2013;70(2):183-90. 10.1001/jamaneurol.2013.646 PMC4574905 23128427

[19] 19. Spruit MA, Wouters EF, Eterman RM, Meijer K, Wagers SS, Stakenborg KH, et al. Task-related oxygen uptake and symptoms during activities of daily life in CHF patients and healthy subjects. Eur J Appl Physiol. 2011;111(8):1679-86. 10.1007/s00421-010-1794-y PMC3156911 21210281

[20] 20. Fernández-Lezaun E, Schumann M, Mäkinen T, Kyröläinen H, Walker S. Effects of resistance training frequency on cardiorespiratory fitness in older men and women during intervention and follow-up. Exp Gerontol. 2017;95:44-53. 10.1016/j.exger.2017.05.012 28526625

[21] 21. Herrero F, Balmer J, San Juan AF, Foster C, Fleck SJ, Pérez M, et al. Is cardiorespiratory fitness related to quality of life in survivors of breast cancer? J Strenght Cond Res. 2006;20(3):535-40. 10.1519/r-18215.1 16977706

[22] 22. Goetz CG, Poewe W, Rascol O, Sampaio C, Stebbins GT, Counsell C, Giladi N, Holloway RG, Moore CG, Wenning GK, Yahr MD, Seidl L; Movement Disorder Society Task Force on Rating Scales for Parkinson’s Disease. Movement Disorder Society Task Force report on the Hoehn and Yahr staging scale: status and recommendations. Mov Disord. 2004;19(9):1020-8. 10.1002/mds.20213 15372591

[23] 23. Skinner JS, McLellan TM. The transition from aerobic to anaerobic metabolism. Res Q Exerc Sport. 1980;51(1):234-48. Erratum in: Res Q Exerc Sport. 2013;84(2):273. 10.1080/02701367.1980.10609285 7394286

[24] 24. Wasserman K, Hansen JE, Sue DY, Whipp BJ. Principles of exercise testing and interpretation. 2nd ed. Philadelphia (PA): Lea & Febiger; 1994. p. 112-31.

[25] 25. Kanegusuku H, Queiroz AC, Chehuen MR, Costa LA, Wallerstein LF, Mello MT, et al. Strength and power training did not modify cardiovascular responses to aerobic exercise in elderly subjects. Braz J Med Biol Res. 2011;44(9):864-70. 10.1590/s0100-879x2011007500100 21845341

[26] 26. Brown LE, Weir JP. ASEP procedures recommendation I: accurate assessment of muscular strength and power. J Exerc Physiol Online. 2001;4(3):1-21.

[27] 27. Cohen J. Statistical power analysis for the behavioral sciences. New York: Routledge. 1988. p. 29-35.

[28] 28. Silva-Batista C, Mattos EC, Corcos DM, Wilson JM, Heckman CJ, Kanegusuku H, et al. Resistance training with instability is more effective than resistance training in improving spinal inhibitory mechanisms in Parkinson’s disease. J Appl Physiol. 2017;122(1):1-10. 10.1152/japplphysiol.00557.2016 27834670

[29] 29. Helgerud J, Thomsen SN, Hoff J, Strandbråten A, Leivseth G, Unhjem R, et al. Maximal strength training in patients with Parkinson’s disease: impact on efferent neural drive, force-generating capacity, and functional performance. J Appl Physiol (1985). 2020;129(4):683-90. 10.1152/japplphysiol.00208.2020 32790593

[30] 30. David FJ, Rafferty MR, Robichaud JA, Prodoehl J, Kohrt WM, Vaillancourt DE, et al. Progressive resistance exercise and Parkinson’s disease: a review of potential mechanisms. Parkinsons Dis. 2012;2012:124527. Review. 10.1155/2012/124527 PMC3236435 22191068

[31] 31. Gray WK, Hildreth A, Bilclough JA, Wood BH, Baker K, Walker RW. Physical assessment as a predictor of mortality in people with Parkinson’s disease: a study over 7 years. Mov Disord. 2009;24(13):1934-40. 10.1002/mds.22610 19672988

[32] 32. Katzel LI, Sorkin JD, Macko RF, Smith B, Ivey FM, Shulman LM. Repeatability of aerobic capacity measurements in Parkinson disease. Med Sci Sports Exerc. 2011;43(12):2381-7. 10.1249/MSS.0b013e31822432d4 PMC3701959 21606869

[33] 33. Stanley RK, Protas EJ, Jankovic J. Exercise performance in those having Parkinson’s disease and healthy normals. Med Sci Sports Exerc. 1999; 31(6):761-6. 10.1097/00005768-199906000-00001 10378900

[34] 34. Uc EY, Doerschug KC, Magnotta V, Dawson JD, Thomsen TR, Kline JN, et al. Phase I/II randomized trial of aerobic exercise in Parkinson disease in a community setting. Neurology. 2014;83(5):413-25. 10.1212/WNL.0000000000000644 PMC4132568 24991037

[35] 35. Kelly NA, Ford MP, Standaert DG, Watts RL, Bickel CS, Moellering DR, et al. Novel, high-intensity exercise prescription improves muscle mass, mitochondrial function, and physical capacity in individuals with Parkinson’s disease. J Appl Physiol (1985). 2014;116(5):582-92. 10.1152/japplphysiol.01277.2013 PMC4073951 24408997

