# Supplementary material for: Effects of resistance training on metabolic and cardiovascular responses to a maximal cardiopulmonary exercise test in Parkinson`s disease
Source: Einstein (Sao Paulo). 2021 Apr 9;19:eAO5940. doi: 10.31744/einstein_journal/2021AO5940 (PMC8051939; doi:10.31744/einstein_journal/2021AO5940)
Supplement: Translation

# Translation: Efeitos do treinamento resistido sobre as respostas metabólicas e cardiovasculares ao teste de esforço cardiopulmonar máximo na doença de Parkinson

**Authors:** Hélcio Kanegusuku, Tiago Peçanha, Carla Silva-Batista, Roberto Sanches Miyasato, Natan Daniel da Silva, Marco Túlio de Mello, Maria Elisa Pimentel Piemonte, Carlos Ugrinowitsch, Cláudia Lúcia de Moraes Forjaz

**Affiliations:** 1 São PauloSP Brasil Hospital Israelita Albert Einstein, São Paulo, SP, Brasil.; 2 São PauloSP Brasil Universidade de São Paulo, São Paulo, SP, Brasil.; 3 Belo HorizonteMG Brasil Universidade Federal de Minas Gerais, Belo Horizonte, MG, Brasil.

**Keywords:** Transtornos parkinsonianos, Anormalidades cardiovasculares, Consumo de oxigênio, Teste de esforço, Treinamento de resistência

## Abstract

**Objetivo:**

Avaliar os efeitos do treinamento resistido nas respostas metabólicas e cardiovasculares ao teste de esforço cardiopulmonar máximo em pacientes com doença de Parkinson.

**Métodos:**

Vinte e quarto pacientes com doença de Parkinson (estágios 2 a 3 de Hoehn e Yahr modificado) foram aleatoriamente randomizados em dois grupos: Controle e Treinamento Resistido. O Grupo Treinamento Resistido realizou, duas vezes por semana, cinco exercícios resistidos, duas a quatro séries, seis a 12 repetições máximas por série. O Grupo Controle manteve seu estilo de vida. No início e após 12 semanas, consumo de oxigênio, pressão arterial sistólica e frequência cardíaca foram avaliados em repouso e durante um teste de esforço cardiopulmonar máximo realizado em um cicloergômetro. As avaliações durante o exercício foram realizadas nas intensidades submáximas (a inclinação da regressão linear entre as variáveis fisiológicas e as cargas absolutas), nas intensidades submáximas relativas (limiar anaeróbico e ponto de compensação respiratória) e na intensidade máxima (pico do exercício). Adicionalmente, foi avaliada a força muscular.

**Resultados:**

Comparado com o início, o consumo de oxigênio pico aumentou, de forma semelhante, em ambos os grupos após 12 semanas. A frequência cardíaca e a pressão arterial sistólica avaliadas nas intensidades submáximas absolutas e relativas, assim como no pico do exercício, não se modificaram em nenhum dos grupos. Finalmente, diferente do Grupo Controle, a força muscular aumentou no Grupo Treinamento Resistido após 12 semanas.

**Conclusão:**

Em pacientes com doença de Parkinson sem comorbidades cardiovasculares, o treinamento resistido aumenta a força muscular, mas não modifica as respostas metabólicas e cardiovasculares ao teste de esforço cardiopulmonar máximo.

## INTRODUÇÃO

A doença de Parkinson (DP) é um distúrbio neurodegenerativo caracterizado pela disfunção do sistema dopaminérgico nigroestriatal e por sintomas motores, como bradicinesia, tremor em repouso, rigidez e instabilidade postural.^(^^1^^,^^2^^)^ Além de sintomas motores, os pacientes também tendem a apresentar disfunções metabólicas e cardiovasculares em repouso^(^^3^^–^^6^^)^ ou durante eventosestressantes, como o exercício.^(^^7^^–^^10^^)^ Respostas metabólicas e cardiovasculares atenuadas durante teste cardiopulmonar com exercício em intensidades submáxima e máxima foram recentemente relatadas em pacientes com DP.^(^^7^^)^ Respostas atenuadas ao exercício podem aumentar o risco cardiovascular nesses pacientes.^(^^11^^,^^12^^)^ Daí a importância de se investigarem estratégias que possam compensar essas limitações.

O treinamento resistido (TR) é recomendado em pacientes com DP para melhorar a força muscular, a funcionalidade (capacidade de andar, por exemplo) e a qualidade de vida.^(^^13^^)^ Sabe-se que o TR também melhora a disfunção autonômica cardíaca no repouso e em situações de estresse (estresse ortostático, por exemplo) nessa população.^(^^14^^)^ Esses achados sugerem que o TR também pode melhorar as respostas cardiovasculares induzidas pelo exercício. Entretanto, essa hipótese ainda não foi confirmada.

O TR melhora o consumo máximo de oxigênio (VO_2max_) em pacientes que padecem de outras condições, como limitações cognitivas, hemiparesia e outras.^(^^15^^,^^16^^)^ Entretanto, nos pacientes com DP em particular, o TR não induziu alterações do VO_2max_,^(^^17^^,^^18^^)^ e os efeitos do TR carecem de confirmação. Entretanto, a avaliação das respostas induzidas pelo exercício submáximo, isto é, respostas obtidas com cargas de trabalho absolutas submáximas e em limiares ventilatórios, é ainda mais relevante nesses pacientes, visto que tais respostas espelham a intensidade de esforço envolvida nas atividades diárias.^(^^19^^)^ Melhoras das respostas ao exercício submáximo foram relatadas por Fernández-Lezaun et al., em idosos sem DP após o TR.^(^^20^^)^ A melhora das respostas cardiovasculares relacionadas ao TR submáximo pode contribuir para a qualidade de vida desses pacientes.^(^^21^^)^ Apesar da relevância do assunto, até onde se sabe, os efeitos do TR sobre as respostas ao exercício submáximo ainda não foram investigados em pacientes com DP.

Este estudo foi idealizado para testar a hipótese de que o TR melhora as respostas submáximas durante o teste de esforço cardiopulmonar máximo em pacientes com DP, sem alterar parâmetros metabólicos (VO_2_) e cardiovasculares (frequência cardíaca – FC e pressão arterial sistólica – PAS) máximos.

## OBJETIVO

Avaliar os efeitos do treinamento resistido nas respostas metabólicas e cardiovasculares ao teste de esforço cardiopulmonar máximo em pacientes com doença de Parkinson.

## MÉTODOS

### Participantes

Os voluntários foram recrutados na Associação Brasil Parkinson. Foram adotados os seguintes critérios de inclusão: mais de 50 anos de idade, estágios 2 a 3 na escalamodificada de Hoehn e Yahr;^(^^22^^)^ ausência de outros distúrbios neurológicos, hipertensão ou diagnóstico de doença cardiovascular; sem uso de medicações com possibilidade de efeito cardiovascular direto, exceto as necessárias para o tratamento da DP e sem limitações quanto à prática do TR. Os pacientes que precisaram de alteração de tipo ou dosagem das medicações tomadas regularmente durante o estudo, envolveram-se em outros programas de exercício físico ou tiveram presença em menos de 80% das sessões de TR foram excluídos.

Este artigo foi baseado em dados extraídos de um estudo maior aprovado pelo Comitê de Ética da Faculdade de Educação Física e Esporte da Universidade de São Paulo (2011/42) e registrado no Registro Brasileiro de Ensaios Clínicos (RBR-5YC53K). Todos os pacientes leram e assinaram um termo de consentimento informado antes da inclusão. Os achados da análise dos efeitos de TR sobre condições de repouso foram publicados.^(^^14^^)^ Este artigo aborda especificamente as repostas durante o exercício.

### Desenho experimental

Este estudo prospectivo randomizado controlado com desenho paralelo foi realizado na Faculdade de Educação Física e Esporte da Universidade de SãoPaulo entre fevereiro de 2012 e março de 2015. Os pacientes com DP foram alocados aleatoriamente para um de dois grupos: Grupo Controle (GC) ou GrupoTreinamento Resistido (GTR), conforme descrição prévia.^(^^14^^)^

As respostas fisiológicas durante teste de esforço cardiopulmonar máximo foram avaliadas no início do estudo e após 12 semanas. No Grupo TR, as avaliações pós-intervenção foram realizadas passadas pelo menos 48 horas da última sessão de treinamento. Os pacientes foram avaliados sob efeito da medicação (*on state*).

### Procedimentos experimentais

Os procedimentos envolvidos no teste de esforço cardiopulmonar máximo foram detalhados em outra publicação.^(^^7^^)^ Os testes foram realizados em um cicloergômetro (Lode, Corival, Países Baixos), por um médico com larga experiência (mais de 10 anos) em teste de exercício máximo. Os pacientes foram familiarizados com o cicloergômetro exercitando-se a uma intensidade confortável por 2 a 3 minutos, 15 a 20 minutos antes do teste, descansando até a normalização dos parâmetros cardiovasculares, e foram testados em seguida. Protocolos individualizados com inclinação foram selecionados para induzir a fadiga entre 8 e 12 minutos após o início do teste. Os protocolos envolveram incrementos de 3 a 15 *watts* por minuto, de acordo com a gravidade da DP e o nível de condicionamento físico. A frequência de pedalagem variou de 50 a 60rpm. Os testes eram interrompidos sempre que os participantes se mostravam incapazes de manter a frequência de pedalagem. Os mesmos incrementos de inclinação foram empregados na avaliação inicial e na avaliação na 12ᵃ semana.

A FC foi registrada a intervalos de 30 segundos, por meio de ecocardiograma de 12 derivações (CardioPerfect^®^, ST 2001, Países Baixos). A pressão arterial auscultatória foi mensurada a cada 2 minutos, por meio de um esfigmomanômetro de mercúrio, por um técnico que desconhecia a alocação dos grupos. A troca de gases respiratórios foi mensurada por meio de análise respiração a respiração, empregando-se um analisador metabólico (Medical Graphics Corporation, CPX/D, Estados Unidos) e calculando-se a média dos dados coletados a intervalos de 30 segundos.

Frequência cardíaca, PAS e VO_2max_ máximos foram definidos como os valores mais altos obtidos durante a fase de exercício do teste e representaram as respostas máximas obtidas no teste. As respostas a intensidades submáximas relativas foram avaliadas nos limiares ventilatórios (isto é, limiar anaeróbico (LA) e ponto de compensação respiratório – PCR)^(^^23^^,^^24^^)^ determinados por dois especialistas independentes, sendo as discrepâncias resolvidas por consenso. As respostas a intensidades submáximas absolutas foram avaliadas comparando-se a inclinação da linha de regressão linear entre os parâmetros fisiológicos (valores de VO_2_, FC e PAS) registrados no início do estudo e após 12 semanas de treinamento.^(^^25^^)^ A regressão foi baseada nos valores mensurados durante o exercício. As inclinações (INCL) das linhas de regressão individuais foram empregadas na análise.

### Intervenções

O programa de TR consistiu em duas sessões de exercício por semana, com período de repouso de 3 a 4 dias entre ambas. As sessões de treinamento foram supervisionadas por um treinador experiente. O programa de TR incluiu exercícios resistidos para membro inferior (*leg press* horizontal, agachamento e panturrilha na máquina) e superior (puxada costas-peito/*lat pulldown* e supino vertical convergente). A carga de treinamento aumentou progressivamente de duas para quatro séries de seis a 12 repetições máximas (RM), conforme descrição prévia.^(^^14^^)^ Foram empregados os seguintes incrementos: primeira e segunda semanas, duas séries de 10-12 RM; terceira e quarta semanas, três séries de 10-12 RM; quinta e sexta semanas, três séries de 8-10 RM; sétima à décima semana, quatro séries de 8-10 RM; 11ᵃ e 12ᵃ semanas, quatro séries de 6-8 RM. O período de descanso entre os exercícios e séries foi de 2 minutos. A carga de trabalho aumentou progressivamente ao longo do período de intervenção. Os incrementos de carga eram introduzidos sempre que os pacientes conseguiam executar duas sessões consecutivas com a mesma carga.

Os pacientes do GC foram orientados a manter o estilo de vida usual durante o período experimental, tendo sido avaliados somente no início do estudo e na 12ᵃ semana.

### Força muscular

A eficácia do TR foi estimada com base na força muscular. Após duas sessões de familiarização com intervalo de 48 horas, a força muscular foi avaliada por meio do teste de uma RM no *leg press*, conforme o protocolo de Brown et al.,^(^^26^^)^ e descrição prévia.^(^^14^^)^ A força muscular foi reavaliada após 12 semanas.

### Análise estatística

A normalidade e a homogeneidade dos dados foram testadas empregando-se os testes de Shapiro-Wilk e Levene, respectivamente. Os dados foram submetidos à transformação logarítmica conforme necessário (isto é, FC_MAX_, FC_INCL_ e VO_2INCL_). As características dos pacientes foram comparadas entre os grupos por meio do teste *t* ou do teste do χ^2^. A análise de variância (ANOVA) de duas vias com desenho misto, adotando-se grupo (GC ou GTR) como fator de variação “entre grupos” e o tempo (início e 12 semanas) como fator de variação “intragrupos” foi realizada para avaliar os efeitos do TR. Nos casos em que os valores F foram significantes, o teste *post-hoc* de Newman-Keuls foi empregado para comparações múltiplas. A PAS que diferiu entre os grupos na análise inicial foi submetida à análise de covariância (ANCOVA), adotando-os valores iniciais como covariáveis. O tamanho do efeito (TE) foi calculado para cada desfecho empregando-se o d de Cohen.^(^^27^^)^ O TE foi classificado como pequeno (TE≤0,49), médio (TE 0,50 a 0,79) ou grande (TE≥0,80). O nível de significância adotado foi de p<0,05. Os dados foram expressos como médias±desvio-padrão.

## RESULTADOS

Assinaram o termo de consentimento informado 44 pacientes com DP. Destes, 30 foram alocados aleatoriamente para o GTR (n=15) ou o GC (n=15). Quatro pacientes do GC e dois do GTR se desligaram do estudo durante o período de intervenção. Ao todo, 24 pacientes completaram o estudo (11 e 13 pacientes do GC e GTR, respectivamente) e tiveram seus dados analisados (Figura 1).

**Figura 1:** Fluxograma dos participantes

As características dos pacientes não diferiram entre os grupos no início do estudo (Tabela 1). Os pacientes do GTR completaram mais de 90% das sessões de treinamento. A força muscular dos membros inferiores se manteve inalterada no GC (90±26kg *versus* 82±26kg), mas aumentou de forma significante no GTR (90±24kg *versus* 108±29kg) após 12 semanas de TR. A força muscular diferiu de forma significante entre os grupos após 12 semanas (F_[1,22]_ = 83,159; p<0,01).

**Tabela 1:** Características dos pacientes com doença de Parkinson alocados para Grupo Controle ou Grupo Treinamento Resistido

		Grupo Controle (n=11)	Grupo Treinamento Resistido (n=13)	Valor de p
Características físicas					
	Sexo masculino/feminino	8/3	11/2	0,48
	Idade, anos	62±9	67±8	0,12
	índice de massa corporal, kg/m²	27,1±3,4	25,9±3,6	0,43
Características da DP					
	Duração da doença, anos	9±4	9±4	0,94
	Hoehn e Yahr modificado, Estágio 2/2,5/3	5/2/4	6/2/5	0,98
Uso de medicações, n					
	Levodopa/carbidopa	11	11	0,18
	Agonista da dopamina	6	5	0,43
	Amantadina	4	6	0,63
	Selegilina	2	3	0,77

Resutlados expressos por n ou média±desvio-padrão.

DP: doença de Parkinson.

Os valores iniciais de consumo de oxigênio não diferiram entre os grupos. Entretanto, ambos os grupos apresentaram aumento significante semelhante de VO_2max_ após a intervenção (F_[1, 22]_ = 0,0338; p=0,86 e p<0,01, efeito da interação e efeito do tempo, respectivamente) (Tabela 2). O VO_2_, o LA e o PCR de repouso também foram semelhantes entre os grupos no início do estudo e se mantiveram inalterados ao longo do tempo. Analogamente, o aumento do VO_2max,_ de acordo com o incremento da carga de trabalho (inclinação da linha de regressão entre VO_2_ e carga de trabalho), foi semelhante entre os grupos no início do estudo e se manteve inalterado ao longo do tempo.

**Tabela 2:** Respostas metabólicas e cardiovasculares avaliadas em repouso e nas intensidades submáxima e máxima durante o teste de esforço cardiopulmonar realizado no início do estudo e após 12 semanas em pacientes com doença de Parkinson alocados para o Grupo Treinamento Resistido ou Grupo Controle

	Grupo Controle		TE	Grupo Treinamento Resistido		TE	Valores F	Valor de p
	Inicial	12 semanas		Inicial	12 semanas			
Carga máxima de trabalho, *watts*	107±54	107±58	0,01	93±37	96±38	0,09	F_[1,22]_=0,5877	0,45
VO_2INCL_, mL.kg^−1^.min^−1^/*watt*†	-1,03±0,38	−0,86±0,14	0,75	−0,90±0,21	−0,88±0,12	0,05	F_[1,22]_=1,1013	0,31
VO_2LA_, mL.kg.^−1^min^−1^	9,7±2,1	9,7±2,2	0,00	10,3±2,3	10,3±2,8	0,02	F_[1,22]_=0,0090	0,93
VO_2PCR_, mL.kg.^−1^min^−1^	14,4±3,5	13,8±3,6	0,03	14,6±2,4	15,7±3,0	0,40	F_[1,20]_=1,7885	0,20
VO_2max_, mL.kg.^−1^min^−1^	17,8±5,5	18,6±5,8*	0,14	18,0±4,2	18,8±4,4*	0,20	F_[1,22]_=0,0338	0,86
FC_repouso_, bpm	76±8	76±11	0,06	73±8	72±10	0,16	F_[1,22]_=0,2541	0,62
FC_INCL’_, bpm/*watt*†	−0,28±0,19	−0,24±0,29	0,18	−0,38±0,17	−0,47±0,26	0,44	F_[1,22]_=3.3466	0,08
FC_LA_, bpm	110±13	108±12	0,17	98±17	99±17	0,07	F_[1,22]_=0,6767	0,42
FC_PCR_, bpm	126±14	124±15	0,13	114±22	118±22	0,19	F_[1,20]_=2,0427	0,17
FC_max_, bpm†	2,16±0,06	2,15±0,06	0,24	2,10±0,09	2,10±0,10	0,03	F_[1,22]_=2,3841	0,14
PAS_repouso_, mmHg	113±13	107±12	0,47	122±18#	119±14#	0,16	F_[1,22]_=0,1535	0,70
PAS_INCL_, mmHg/*watt*	0,72±0,64	0,95±0,80	0,33	0,45±0,25	0,57±0,25	0,50	F_[1,18]_=0,4273	0,52
PAS_LA_, mmHg	139±17	132±15	0,41	137±16	144±19	0,43	F_[1,22]_=3,1499	0,09
PAS_PCR_, mmHg	156±22	148±18	0,39	149±15	158±18	0,58	F_[1,20]_=5,6330	0,03
PAS_max_, mmHg	171±23	170±25	0,10	160±20	167±20	0,37	F_[1,22]_=1,6963	0,21

Resultados expressos por média±desvio padrão.

* diferença significante em relação ao valor inicial (p<0,05);

† logaritmo;

# diferença significante em relação ao Grupo Controle (p<0,05).

TE: tamanho do efeito; VO_2_: consumo de oxigênio; INCL: inclinação; LA: limiar anaeróbico; PCR: ponto de compensação respiratória; FC: frequência cardíaca; PAS: pressão arterial sistólica.

A FC mensurada durante o repouso, no LA, no PCR e na intensidade máxima de exercício não diferiu entre os grupos e se manteve inalterada após 12 semanas, assim como o incremento da inclinação da linha de FC por *watt* durante o teste. Da mesma forma, a PAS mensurada durante o repouso, no LA, no PCR e na intensidade máxima de exercício, não diferiu entre os grupos e se manteve inalterada após 12 semanas, assim como o incremento da inclinação da linha de PAS por *watt* durante o teste.

## DISCUSSÃO

O principal achado deste estudo foi que o TR aumentou a força muscular nos membros inferiores, mas não afetou as respostas metabólicas e cardiovasculares obtidas em intensidades máximas ou submáximas relativas e absolutas durante o teste de esforço cardiopulmonar máximo em pacientes com DP.

O aumento da força muscular nos membros inferiores no GTR em relação ao GC denota a eficácia do protocolo de treinamento empregado neste estudo. Achados semelhantes foram relatados em estudos anteriores,^(^^17^^,^^18^^)^ nos quais a força muscular dos membros inferiores também aumentou após o TR. Alterações neuroplásticas no córtex motor primário e adaptações neurais ao TR podem explicar o ganho inicial de força nos membros inferiores nesses pacientes.^(^^28^^–^^30^^)^ Tais ganhos de força podem ter relevância clínica nessa população, visto que a perda de massa muscular e força se correlacionam com pior qualidade de vida em pacientes com DP.^(^^31^^)^

O aumento significante do VO_2max_ após 12 semanas em ambos os grupos indica que a melhora não pode ser atribuída ao programa de TR. Na verdade, o aumento observado no GC sugere um efeito de aprendizado proporcionado pela repetição dos teste. Um estudo prévio^(^^32^^)^ com pacientes com DP submetidos a três testes de esforço cardiopulmonar máximo em dias diferentes revelou aumento significante do VO_2max_ entre o primeiro e o segundo teste. Efeitos semelhantes foram observados nos pacientes analisados deste estudo.

A ausência de efeito do TR sobre o VO_2max_ reflete os resultados de outros estudos que também não encontraram alterações do VO_2max_ em resposta ao TR em pacientes com DP.^(^^17^^,^^18^^)^ Esses achados coincidem com os de um estudo prévio realizado por este grupo de pesquisa,^(^^25^^)^ no qual o VO_2max_ se manteve inalterado em idosos saudáveis após 16 semanas de TR e de potência. Em contrapartida, estudos realizados com outras populações de pacientes adultos com comprometimento neurológico ou condições neurológicas crônicas (limitações cognitivas ou hemiparesia crônica) relataram aumento do VO_2max_ após o TR.^(^^15^^,^^16^^)^ Essa discrepância pode refletir diferenças de alterações patofisiológicas entre doenças neurológicas distintas e o respectivo impacto sobre a resposta dos pacientes ao TR. Portanto, seria cabível dizer que, pelo menos em pacientes com DP leve a moderada, o TR não afeta o VO_2max_.

Um diferencial deste estudo foi a investigação de parâmetros de exercício submáximo menos influenciados pelas incapacidades motoras associadas à DP.^(^^33^^)^ Para que isso fosse possível, os pacientes foram avaliados nas intensidades máximas e submáximas de exercício. As respostas à intensidade submáxima absoluta foram avaliadas com base nas alterações metabólicas e cardiovasculares induzidas pelos incrementos de carga de trabalho, enquanto as respostas referentes à intensidade submáxima relativa foram avaliadas no LA e no PCR. Contrariando a hipótese, o TR não teve influência sobre o VO_2_, a FC ou a PAS nas intensidades submáximas absoluta e relativa. Esse achado reflete os de estudo anterior que avaliou respostas ao TR de intensidade submáxima absoluta e relativa em indivíduos sem DP.^(^^25^^)^ Portanto, apesar da seleção de parâmetros que sofrem menos influência das limitações relacionadas à doença, este estudo não demonstrou impactos significantes do TR sobre as respostas obtidas durante o teste progressivo de exercício.

Respostas metabólicas e cardiovasculares superiores durante o teste de esforço cardiopulmonar máximo se traduzem em melhor qualidade de vida em pacientes com DP. Tais respostas foram obtidas com treinamento aeróbico nessa população.^(^^18^^,^^34^^)^ Ainda assim, o TR pode melhorar a força muscular, a funcionalidade (capacidade de andar, por exemplo) e a qualidade de vida em pacientes com DP.^(^^17^^,^^18^^,^^26^^,^^28^^,^^29^^,^^33^^,^^35^^)^ Os achados deste estudo corroboram a recomendação de programas de treinamento que agregam exercícios aeróbicos e de força para pacientes com DP leve a moderada.

### Limitações do estudo

Este estudo tem algumas limitações. A exclusão de pacientes com diagnóstico de hipertensão ou doença cardiovascular limitou o tamanho da amostra. Entretanto, a exclusão foi considerada necessária para separar os efeitos do TR das alterações metabólicas e cardiovasculares inerentes à DP. Além disso, uma vez que a seleção se limitou aos pacientes nos estágios 2 ou 3 da escala modificada de Hoehn e Yahr, os achados podem não se aplicar a pacientes em estágios diferentes da DP.

## CONCLUSÃO

A prática do treinamento resistido por 12 semanas melhora a força muscular nos membros inferiores, mas não afeta as respostas metabólicas e cardiovasculares obtidas em intensidades máxima ou submáxima durante o teste de esforço cardiopulmonar máximo em pacientes com doença de Parkinson sem comorbidades cardiovasculares.
